# Sex, density dependence, and urbanization level shape host infection by an obligate endoparasite

**DOI:** 10.1371/journal.pone.0340623

**Published:** 2026-02-12

**Authors:** J. Scott MacIvor, Thomas C. K. Hall

**Affiliations:** 1 Department of Biological Sciences, University of Toronto Scarborough, Toronto, Ontario, Canada; 2 Ecology and Evolutionary Biology, University of Toronto, Toronto, Ontario, Canada; 3 Department of Zoology, Faculty of Science, Charles University, Prague, Czech Republic; USDA Agricultural Research Service, UNITED STATES OF AMERICA

## Abstract

Anthropogenic changes alter host–parasite dynamics, but the way urbanization influences these relationships remains understudied, despite the diversity in species and transmission modes. We investigated infection of the solitary wasp *Isodontia mexicana* Saussure, 1867 (Hymenoptera: Sphecidae) by the twisted-wing insect *Eupathocera* auripedis Pierce, 1911 (Strepsiptera: Xenidae), a rarely documented obligate endoparasite, over a three-year period. We recorded 40 stylopized *I. mexicana* individuals out of 321 wasps examined, totaling 69 individual *E. auripedis*, including six found embedded in a single host. Female wasps were larger than males and showed no change in body size with stylopization, whereas stylopized males were significantly smaller than their non-stylopized counterparts. We observed density dependence between host and parasite: wasp abundance, stylopization rates, and the number of strepsipterans per host were all positively correlated. All variables declined significantly along a gradient from low to high urbanization, with tree cover the most important determinant for nesting habitat quality. Although open green space was not directly associated with host or parasite variables, it remains important for *I. mexicana*, which depends on these areas for tree crickets (*Oecanthus* spp.) to provision offspring and nectar from asters and mints. Thus, while nesting may be more dependent on forested areas, these highly mobile wasps likely rely on open green spaces for foraging which may serve as interception points for parasites. Further research is needed to better understand the influence of land cover on host–parasite interactions. Our findings highlight the utility of trap nests for improving the study of cryptic interactions, the use of stylopization as a bioindicator, and new insights into urban strepsipteran ecology.

## Introduction

Urbanization alters biological communities, including host–parasite dynamics. Parasite presence depends on host abundance, which, in turn, is shaped by urban-modified habitat conditions. In urban areas, declining or isolated populations may experience reduced parasite pressure, resulting in enemy release [1]. Conversely, urban-tolerant hosts can facilitate parasite transmission if parasites are similarly tolerant. For example, the prevalence of the parasite *Crithidia bombi* Lipa & Triggiani, 1988 (Trypanosomatida: Trypanosomatidae) was significantly higher in *Bombus vosnesenskii* Radoszkowski, 1862 (Hymenoptera: Apidae) in gardens surrounded by greater proportions of impervious urban cover [2]. Thus, cities may sustain host–parasite interactions, particularly within remnant green spaces (e.g., forests, grasslands), or in novel habitats such as green roofs and community gardens.

Parasites affect hosts in diverse ways. While lethal infections limit parasite dispersal, particularly in fragmented host populations, sublethal effects may permit parasite persistence and dispersal, especially in mobile hosts [3]. These interactions can serve as indicators of habitat quality. For example, Sheffield et al. [4] proposed kleptoparasites as indicators of environmental quality, and this may extend to rarer parasite associations that require more specific methods to detect.

While surveying cavity-nesting bees and wasps using trap nests [5], a common inhabitant was the grass-carrying wasp, *Isodontia mexicana* Saussure 1867 (Hymenoptera: Sphecidae). During the emergence period from trap nests, we observed numerous stylopized *Isodontia mexicana* individuals (i.e., parasitized by a strepsipteran endoparasite; a parasite that lives within the host), identified as *Eupathocera auripedis* [6] (Strepsiptera: Xenidae) (Jakub Straka, pers. comm.). Xenidae infect wasps in the families Crabronidae, Bembicidae, Sphecidae, and Vespidae [7,8] and cause sublethal effects, allowing hosts to continue flying, mating, foraging, and nesting, thereby aiding parasite transmission. Like most strepsipterans, *E. auripedis* is rarely observed on the wing; the first, mobile larval stage, called the triungulin, intercepts foraging female wasps and are transported to the nest where they infect the developing offspring [9]. Upon reaching maturity, adult male strepsipterans emerge from the host at nesting sites or resource-rich areas to seek mates. These mates are neotenic females that never develop an adult insect body and are permanently embedded in their host [10,11]. Successful reproduction leads to the release of new triungulins that await contact with another foraging female host, continuing the cycle. Species like *I. mexicana* that are infected by *E. auripedis*, and readily use artificial trap nests to provision offspring, represent an accessible model system to investigate the ecological relationships mediating these rarely studied host-parasite interactions.

Despite more than a century of natural history observations of strepsipterans (e.g., [12,13,6,14]), research on taxonomy and systematics have been the main focus in the past few decades [7] with only a handful of studies based on their ecology. For instance, Hrabar et al. [10] described the life history of *Xenos peckii* Kirby, 1802 (Xenidae: Strepsiptera) in *Polistes fuscatus* (Fabricius, 1793) (Vespidae: Hymenoptera), noting vertical transmission during provisioning and phoretic dispersal. Short-lived male strepsipterans seek out females residing in host abdomens at nesting sites or resource-rich areas [10,11]. Despite these advances, key aspects of strepsipteran ecology, including natural history and stylopization dynamics, remain poorly understood. Most studies rely on preserved specimens from field netting, or malaise trapping campaigns [15,7], with few trap nest observations (e.g., [16]). Stylopization rates vary by region and species; for example, 11% in *Sphex ichneumoneus* (Linnaeus, 1758) (Hymenoptera: Sphecidae) by *Paraxenos westwoodi* [Templeton, 1841] (Strepsiptera: Xenidae) in Montana [17], and 7–25% in *Polistes dominulus* (Christ, 1791) (Hymenoptera: Vespidae) in Maine [18].

**Fig 1:**
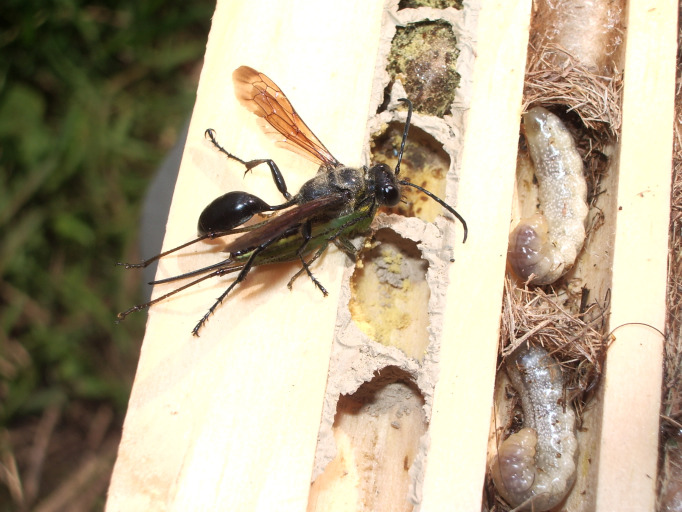
Image of an *Isodontia mexicana* female carrying a tree cricket to provision a nest. Two exposed nests are shown, one of the Blue Orchard Mason bee (*Osmia lignaria*) (left) and *I. mexicana* (right). Photo credit: Peter Hallett.

*Isodontia mexicana* is a solitary wasp that provisions brood with tree crickets from the genus *Oecanthus* (Oecanthidae) [19,20], including *O. nigricornis* Walker, F., 1869 and *O. quadripunctatus* Beutenmüller, 1894 in our study region [21] (Fig 1). These prey species inhabit forest edges and open green spaces dominated by *Solidago*, *Rubus*, and *Daucus*, where females oviposit in stems of various herbaceous plants [22]. Green spaces are also important for *I. mexicana* which partition provisioned brood cells using dried grass, and nectar on flowering plants like Asters (Asteraceae) and mints (Lamiaceae) [23]. Once a suitable nesting site is located, a female typically provisions brood in sequence, supplying female offspring first [19]. Because female larvae require larger prey masses, they are provisioned with more prey items [20], necessitating additional foraging trips that may increase exposure to strepsipteran parasites. Given the strong association between *I. mexicana* and green space, we expected that urbanization would influence both nesting success and parasitism dynamics. Over three years, we monitored *I. mexicana* emerging from trap nests in Toronto, Canada for stylopization. Our objectives were to quantify infection rates, assess sublethal impacts, and determine possible relationship to anthropogenic change.

We first hypothesized that stylopization rates would be higher in female offspring because their larger prey requirements necessitate more maternal foraging trips, increasing exposure to strepsipteran parasites. Second, we hypothesized that infected wasps will be smaller than uninfected, representing a sublethal impact of stylopization [24,25]. Third, given the patchy distribution of strepsipterans [11], we hypothesized a density-dependent relationship between host and parasite, predicting higher infection rates in areas with greater host abundance due to increased encounter probability. Finally, we hypothesize that stylopization declines with urban land cover due to loss of green space and associated resources. To our knowledge, this is the first study to document stylopization rates in an urban setting and to examine how infection rates correspond with land cover gradients.

## Methods

### a. Collections

We set out trap nests to survey cavity-nesting bees and wasps at approximately 200 locations, in four site types: urban parks, gardens, and green roofs, in the city of Toronto, Canada, over a three-year period (2011–2013). The trap nests were made of 30 cardboard tubes of three different inner widths (3.4 mm, 5.5 mm, 7.6 mm) (10 tubes per width) into which bees and wasps provision nests. *Isodontia mexicana* readily nests in trap nests [26,19,5] and exclusively used the largest of the three widths. All trap nests were opened and each individual wasp larva was labelled with a unique ID number, placed in an incubation tray (Corning 24-cell assay tray), and overwintered at 4°C. The individuals were moved to a sealed growth chamber in the April following their collection and artificial overwintering and were kept at 26°C and ~60% humidity until they emerged as adults. As a result of opening all nests and rearing each larva individually, any *E. auripedis* present would have come from the mother’s original provisioning and not from post-collection contamination.

**Fig 2:**
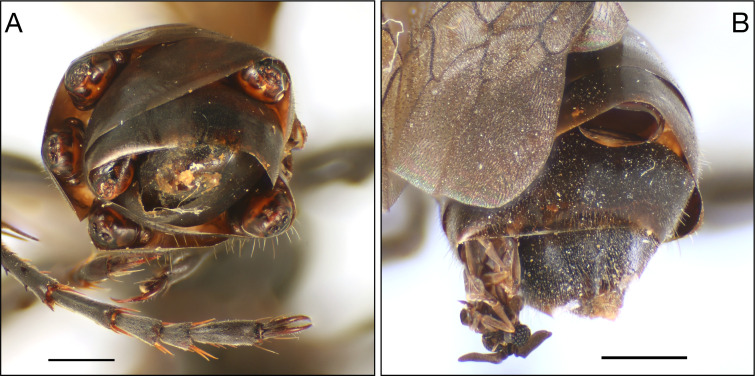
**A.** Stylopized *Isodontia mexicana* with six *Eupathocera auripedis* males embedded within the abdominal segments. Scale line represents 1mm. **B.** Another stylopized *I. mexicana*, showing one male that has eclosed but not yet exited its capsule, along with two empty capsules indicating previously emerged males. Photo credit: Steve Paiero.

The resulting adult *I. mexicana* wasps were pinned, labeled with the individual’s location and date of capture, and curated in the collection of the Biodiversity of Urban Green Spaces (BUGS) lab at the University of Toronto Scarborough. To complete this study, all pinned wasps were reexamined using an Olympus SZ61 microscope to quantify stylopization and body condition. Stylopized *I. mexicana* are identified by the characteristic, bulging extrusions on the abdomen of the wasp (Fig 2A). Each extrusion represented one *E. auripedis* male, and we had evidence of *E. auripedis* males emerging from only one wasp, in which two empty puparia and a partially emerged adult were observed (Fig 2B). No female *E. auripedis* were identified in the pinned specimens because none were visibly extruded and therefore undetectable. To preserve the wasp specimens, we did not dissect hosts to search for them. Of the 358 adults that emerged, we obtained sex, body size, and stylopization data for 321 individuals after excluding damaged specimens or those donated to synoptic collections. Of these, 40 stylopized wasps were identified and were parasitized by 69 individual *E. auripedis* males.

### b. Morphometric measurements

Five morphological traits were measured for both stylopized and non-stylopized wasps. These morphological traits were head width (mm), head length (mm), body length (mm), wing length (mm), and abdomen width (mm) (S1 Table). These traits were individually measured using calipers (0–150 mm, 6-inch caliper, displayed two decimal places).

For stylopized individuals, the number of extrusions was used to count the number of parasitoids present per wasp. The location of each extrusion or empty puparia on the host body was recorded (Fig 2). Location of the individual was defined using three characters; left or right side of the abdomen; tergal or sternal side of the abdomen; and the abdomen segment number above the extrusion, counted from the front of the wasp to the rear.

### c. Land cover classification

We used the land cover data calculated as in Xie et al. [27] from the 2008 Forest and Land Cover dataset (0.6 m raster pixel resolution of seven different cover classes) [28]. We calculated urban land cover as the sum of the proportion of buildings, roads, and other paved surfaces. Additionally, we calculated % open green space (meadows, gardens), and % closed green space (forests). All three were quantified at a 250m radius to test for relationships between land cover and *I. mexicana* infection rates. We could not calculate landscape variables for sites that were not completely contained within the raster dataset, hence two surveyed sites outside the City of Toronto were removed from analysis.

In total, *I. mexicana* were recorded from 52 locations in trap nests over all three years of the study, and morphometric data were obtained from individuals from 35 sites.

### d. Analysis

All analyses were conducted using R statistical software (version 4.3.2; [29]) and collapsed over all three years of study (data used in this study are available in S1 File). First, a Pearson’s Chi-squared test with Yates’ continuity correction was used to compare the occurrence of strepsipterans between male (n = 17) and female *I. mexicana* (n = 23) after individuals of unknown sex were removed. Then, a Welch two sample t-test was used to compare strepsipteran abundance per individual between stylopized males and females. Additionally, generalized linear models (GLMs) were used to assess the effect of site type (predictor) on three response variables: *I. mexicana* abundance, number of stylopized wasps per site, and total number of strepsipterans.

Pairwise Pearson correlation coefficients were computed among the five morphometric variables and were highly correlated using the *corr.test* function in the *psych* package [30] (S2 Table). To account for multicollinearity, each variable was centered to mean zero and scaled to unit variance using z-score standardization and included in a principal component analysis (PCA) using the *prcomp* function in the *stats* package of base R. The first principal component (PC1) captured the dominant axis of body size (77.6% variance explained) with larger PC1 values representing a larger head, body, abdomen and wing length (S3 Table). To simplify analyses, we use PC1 as a single composite size variable. We fit two linear models. The first model tested the effects of sex, strepsipteran occurrence (presence/absence), and their interaction on PC1. The second model tested the same structure, but it replaced strepsipteran occurrence with strepsipteran abundance as the predictor. Site type was not included in these models.

During study, we observed that, *Isodontia mexicana* tended to nest in home gardens and parks disproportionately more than in community gardens and green roofs. We used a logistic regression to assess the probability of stylopization by site type to test this. Estimated marginal means were calculated for comparison using the *emmeans* package [31]. Probabilities of stylopization per site type were obtained by applying the inverse logit transformation to the log-odds estimated in the model.

To compare land cover with the abundance of *I. mexicana*, strepsipteran occurrence, and abundance, we first calculated Pearson correlation coefficients to assess collinearity among these variables (S4 Table). Percent tree cover and percent urban cover were strongly negatively correlated (r = −0.88, p < 0.001). Grass cover was moderately negatively correlated with tree cover (r = −0.39, p = 0.01) and weakly correlated with urban cover (r = −0.10, p = 0.49). These results served as justification to reduce dimensionality, we conducted a principal component analysis (PCA) on standardized values of the three landscape variables (S5 Table; S1 Fig). The first two components (PC1, PC2) explained 100% of the variance and were used as composite landscape predictors in subsequent models. PC1 (63.1%) represented a gradient from urban cover to treed area, with higher values indicating greater tree cover. PC2 (36.9%) represented a gradient of open green space (e.g., grass), with higher values indicating more open green area (S1 Fig).

We used GLMs to test whether *I. mexicana* abundance and stylopization were associated with the land use gradients. We first fit a Poisson GLM and identified overdispersion (S2 Fig) and so to account for this, we modelled *I. mexicana* abundance using a negative binomial GLM (glm.nb, *MASS* package; diagnostic plots: S3 Fig) with PC1, PC2, and site type as predictors and wasp abundance as the response variable.

To test whether site-level strepsipteran occurrence was associated with landscape composition, we fit a binomial GLM with PC1 and PC2 as predictors and strepsipteran occurrence (presence/absence) as the binary response. We also obtained odds ratios (OR) from the model coefficients to assess the odds of strepsipteran occurrence change with each unit increase in landscape variables. Finally, we modelled total strepsipteran abundance using the same procedure as for wasp abundance; by fitting a Poisson GLM first, identifying overdispersion (S4 Fig), and refitting with a negative binomial GLM (S5 Fig).

## Results

**Fig 3:**
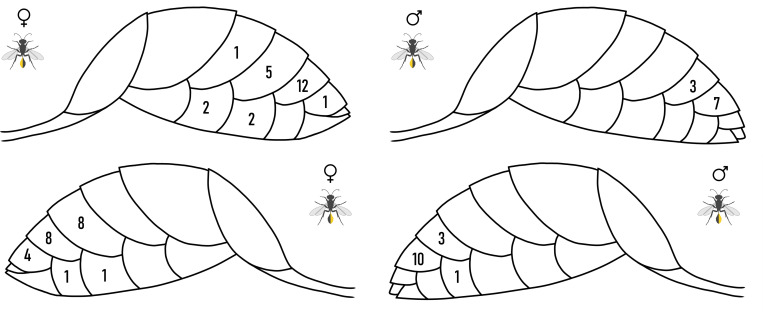
Diagram depicting the sternite and tergite positioning of each strepsipteran discovered across the study. The section of the wasp being viewed is highlighted in yellow on the wasp icon.

From 35 sites where *Isodontia mexicana* had provisioned nests over a three-year period, we inspected adult wasps for stylopization by *Eupathocera auripedis*. We identified 40 stylopized individuals (23 females, 17 males) from 21 sites. In total, 69 individual *E. auripedis* males were recorded. Infected hosts contained between 1 and 6 *E. auripedis* males embedded within the abdomen (see Fig 2A), with most stylopized wasp individuals (55%) harbouring a single strepsipteran. The position of individual strepsipterans varied across tergites and sternites, with 89.8% recorded under tergites (Fig 3). Tergite 3 was the most common location, with 53.6% of all strepsipterans found there (N = 37).

There was no difference between male and female *I. mexicana* in the occurrence of infection (χ² = 0.470, p = 0.49) or in the abundance of strepsipterans per individual (t = 1.63, p = 0.12). Males were significantly smaller in overall body size (PC1) than females (t = −3.39, p = 0.0008). Stylopization had a sex-specific effect: infected males were significantly smaller (p < 0.05) than uninfected males (t = −2.55, p = 0.011), while no significant difference was observed among females (t = 1.42, p = 0.16).

**Fig 4:**
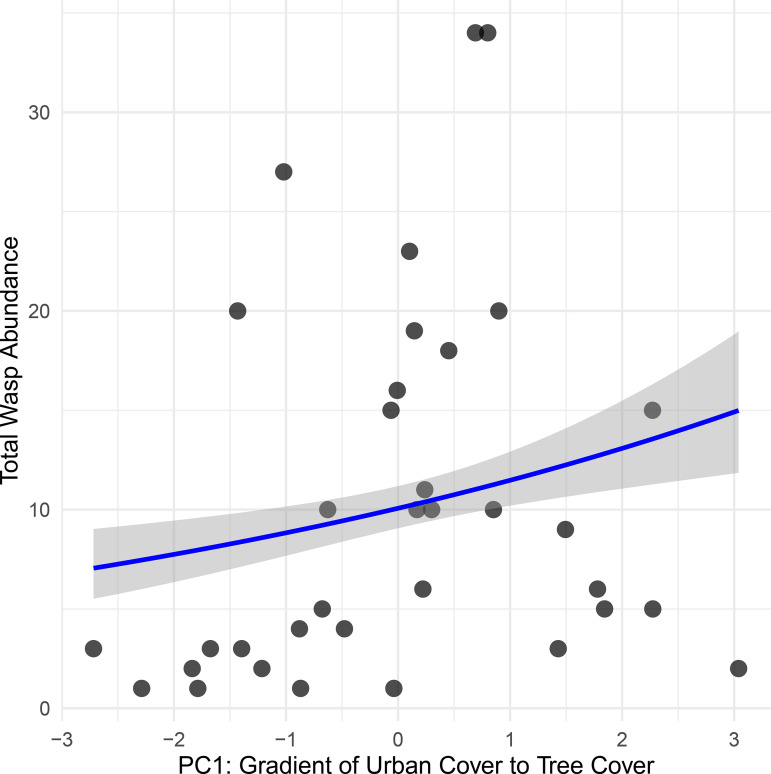
Significant relationship between host *I. mexicana* abundance and PC1 which represents an urbanization gradient where higher values are lower urban cover and higher tree cover.

*Isodontia mexicana* abundance was significantly positively associated with PC1 (z = 3.87, p = 0.0001), indicating that sites with greater tree cover and lower urban cover supported higher wasp abundance (Fig 4). PC2 which represented a gradient of open green space had no significant effect on wasp abundance (z = 1.22, p = 0.22). The estimated marginal means from a logistic regression suggest that the probability of stylopization varies by site type. Community gardens sites had the highest estimated log-odds (−0.56 ± 0.50), corresponding to a predicted probability of ~36%, followed by gardens (~17%), parks (~16%), and roofs (~8.5%). However, sample sizes differed substantially across site types (e.g., gardens had 195 individual *I. mexicana* vs. 7 in community gardens and on green roofs), influencing the precision of these estimates.

**Fig 5:**
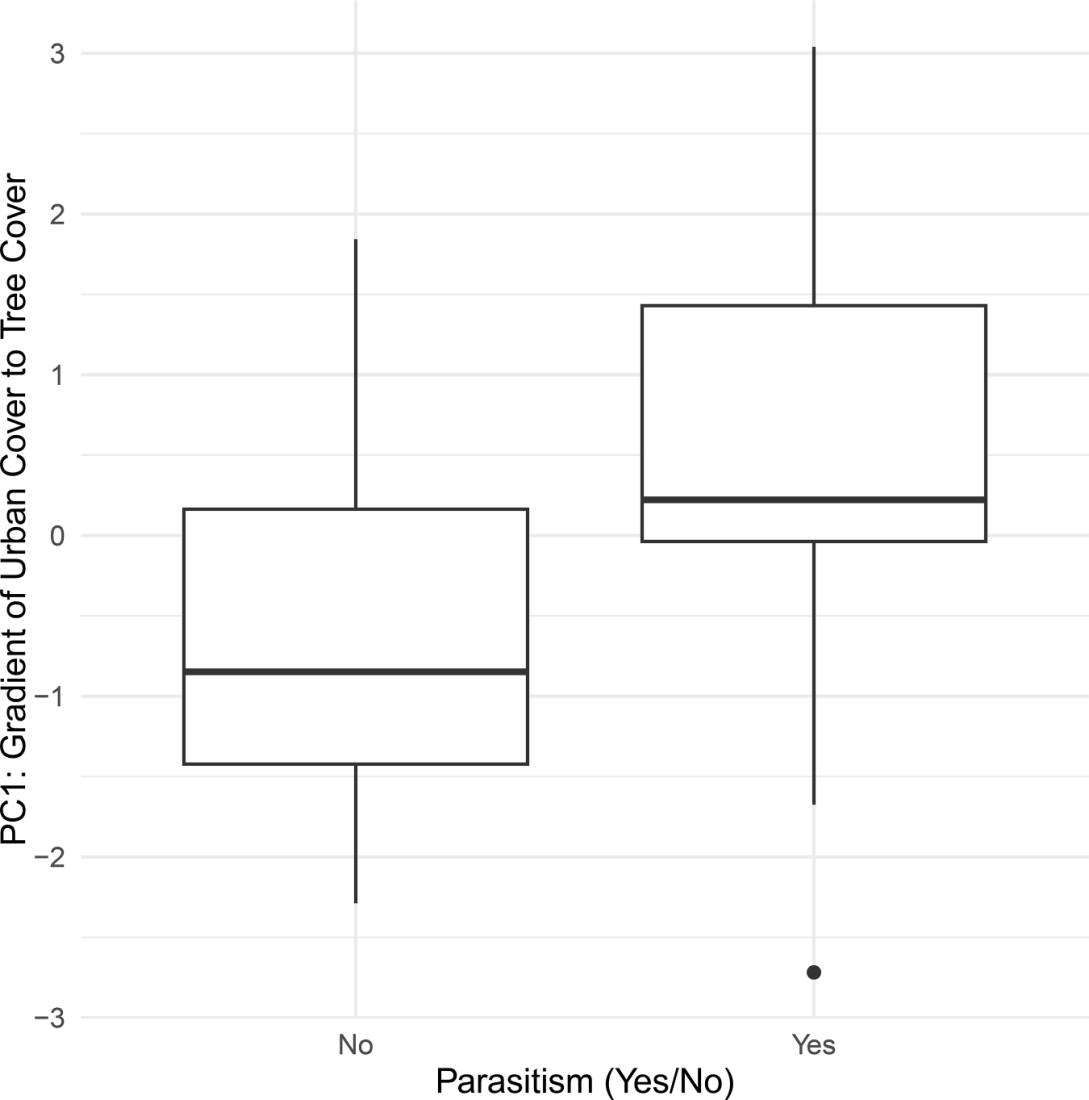
Significant relationship between styplopized and non-stylopized *I. mexicana* individual and PC1 which represents an urbanization gradient where higher values are lower urban cover and higher tree cover.

Strepsipteran occurrence was significantly positively associated with PC1 (z = 2.38, p = 0.017), with sites having greater tree cover exhibiting higher odds of strepsipteran presence (odds ratio ≈ 2.36 per unit increase in PC1) (Fig 5). PC2 again had no significant effect (z = −0.28, p = 0.78). For strepsipteran abundance, there was a positive but marginally significant effect of PC1 (z = 1.89, p = 0.059), while PC2 remained non-significant (z = −1.08, p = 0.28).

## Discussion

By evaluating stylopization of *Isodontia mexicana* and abundance of *Eupathocera auripedis* present in hosts in an urban centre, our study provides insight into how sex, density dependence, and urbanization jointly shape parasitism. We evaluated four hypotheses and conclude that stylopization in *I. mexicana* is sex-specific in fitness costs, density-dependent, and declines with urbanization through effects on habitat quality and host density.

### a. Stylopization is sex-specific *in* fitness costs

First, we found that stylopization was equally likely in both sexes, rejecting our first hypothesis that stylopization rates would be higher in female hosts because they are provisioned with more prey. As female and male *I. mexicana* offspring occur in the same natal nest, infection risk appears to be determined primarily by whether the provisioning female inadvertently introduces triungulin larvae into the nest. Evidence from our study suggests triungulin infect whichever sex is being provisioned at the time of interception.

We find partial support for our second hypothesis, which predicted body size reductions in stylopized hosts. Stylopized males were significantly smaller than uninfected males, whereas no size difference was detected among females. As body size is a key correlate of fitness in solitary wasps, including *I. mexicana* [20], this sex-specific pattern suggests sublethal effects, and that females may better buffer the energetic costs of stylopization. From the perspective of the strepsipteran, maintaining host females in good condition may also be adaptive. Larger females provision nests and may range farther, increasing opportunities for strepsipteran dispersal during flower visitation. In bees, larger body size is associated with greater foraging distance and efficiency [32], and similar relationships likely apply to solitary wasps. Thus, selection may favour stylopization strategies that minimize fitness costs to female hosts, allowing infected females to contribute to strepsipteran transmission dynamics, while smaller, shorter-lived males may experience greater body condition-related developmental constraints and associated behaviour from stylopization. Sex-specific behaviours related to increasing transmission have been documented among parasitized and non-parasitized insects [33]. In *I. mexicana*, this could serve to maintain dispersal via visits to high quality resources such as Asters and mints [23], creating hotspots for infection.

### b. Density-dependent relationship

We found support for our third hypothesis that stylopization rates exhibit host density dependence. With higher *I. mexicana* abundance, there was significantly higher stylopization and marginally higher strepsipteran abundance. This aligns with classical host-parasite models and empirical studies in which parasite transmission scales positively with host density [34,35]. Greater host density likely increases encounter rates between ovipositing strepsipterans and susceptible *I. mexicana* hosts. As *I. mexicana* is widespread in North America, where it is native, and currently expanding throughout Europe [36], it would be interesting to know whether *E. auripedis* co-occurs in its non-native environment and whether the association is as widespread. Within hosts, density dependence is also evident: over half (55%) of infected adults harboured only a single strepsipteran. This pattern suggests that space and resource limitation within the host abdomen constrain the survival of multiple parasites [37], consistent with density-dependent resource competition [38]. With that said, we documented five and six strepsipterans on two individuals, respectively. This represents a level of superparasitism associated with substantial fitness costs and accelerated parasite development in other strepsipteran-host systems [39,40,41].

### c. Land use gradients

We find support for our fourth and final hypothesis that stylopization would decline with increasing urbanization. Stylopization was negatively correlated with urbanization, but our principal coordinate analysis suggests this pattern is better explained by a positive association with tree cover, as indicated by the significant relationship between stylopization and PC1 (which reflects increasing tree cover and decreasing urban cover). It is important to note our results apply specifically to nesting sites, even though *I. mexicana* forage across many different urban habitat types. Tree cover appears to represent high quality environments for nesting in *I. mexicana* [27], however, foraging adults may fly to open green spaces such as pollinator gardens and meadows where tree crickets, nectar resources (asters, mints), and grass used to partition brood cells in nests, are all located. In sum, urbanization did not uniformly disrupt these host-parasite interactions; instead, stylopization was related more to habitat quality and host density.

These findings demonstrate the nuance and context-dependence of the enemy release hypothesis (e.g., [1]) in urban systems. While stylopization declined with urbanization, this pattern reflected habitat-mediated reductions in host wasp density rather than an intrinsic escape from natural enemies. In high quality urban green spaces, host-parasite interactions remained intact, consistent with evidence that urban-tolerant species can sustain parasites where suitable resources persist [42]. Rather than broadly suppressing stylopization, urbanization may result in heterogeneous enemy landscapes, with parasitism structured by resource availability and host density [43,42].

## Conclusions

We show that sex, host density, and habitat gradients interact to structure stylopization in the solitary wasp *I. mexicana*. Our findings contribute to the growing recognition that host-parasite dynamics persist in urban landscapes and are shaped by habitat quality and density-dependent processes. Rarely studied but widespread strepsipterans such as *E. auripedis* may serve as sensitive indicators of hidden ecological interactions. This significance could be spurred by the fact *I. mexicana* is adventive in Europe, raising key questions about parasite co-invasion or enemy escape in novel ranges. Finally, given that strepsipterans can be identified from the abdomen of pinned insects, which was the strategy used in this study, museum collections represent an underutilized resource for reconstructing these cryptic host-parasite histories [44]. Leveraging collections may provide new insights into the temporal dynamics of parasitism in environments undergoing anthropogenic change.

## Supporting information

S1 Table Body size measurement of styplopized and non-stylopized *I. mexicana* and comparisons between male and female morphometrics. (DOCX)

S2 Table Pearson correlation coefficients among morphological traits of *Isodontia mexicana.* All traits show moderate to high positive correlation.
(DOCX)

S3 Table Principal component analysis (PCA) of five morphological traits measured in *Isodontia mexicana.* The top section shows the standard deviation, proportion of variance explained, and cumulative variance for the first five principal components. The lower section lists the loadings (eigenvectors) of each trait on each principal component.
(DOCX)

S4 Table Pearson correlation coefficients among land cover variables within a 250 m buffer around study sites. Tree cover is strongly negatively correlated with urban cover, while grass cover shows weak correlations with both.
(DOCX)

S5 Table Summary of principal component analysis (PCA) results based on three land cover variables within a 250 m buffer. The top section shows the standard deviation, proportion of variance, and cumulative variance explained by each principal component. The lower section shows the loadings (eigenvectors) of each variable on the first three principal components.
(DOCX)

S1 Fig Principal component analysis (PCA) biplot showing variation in landscape cover across sampling sites. (DOCX)

S2 Fig Diagnostic plots for the Poisson GLM testing effects of landscape composition (PC1, PC2) on *I. mexicana* abundance. (DOCX)

S3 Fig Diagnostic plots for the negative binomial GLM refit correcting for overdispersion in *I. mexicana* abundance. (DOCX)

S4 Fig Diagnostic plots for the Poisson GLM testing effects of landscape composition on total wasp abundance. (DOCX)

S5 Fig Diagnostic plots for the negative binomial GLM refit correcting for overdispersion in total wasp abundance. (DOCX)

S1 File Zipped folder of all data used in the study and an accompanying README file. (ZIP)
