# Supplementary material for: Sex, density dependence, and urbanization level shape host infection by an obligate endoparasite
Source: PLoS One. 2026 Feb 12;21(2):e0340623. doi: 10.1371/journal.pone.0340623 (PMC12900303; doi:10.1371/journal.pone.0340623)
Supplement: S1 Table — (DOCX) [file pone.0340623.s001.docx]

Table S1. Body size measurements in mm (except PC1) of styplopized and non-stylopized *I. mexicana* and comparisons between male and female morphometrics.

| **Trait** | **Description** | **Female (No)** | **Female (Yes)** | **Male (No)** | **Male (Yes)** |
| --- | --- | --- | --- | --- | --- |
| **Head width** | Between the outer margins of the compound eyes | 3.55  (2.43–4.44) | 3.66  (2.91–4.20) | 3.40  (2.56–4.12) | 3.17  (2.60–3.60) |
| **Head length** | Anterior margin of the clypeus to the posterior margin of the head | 3.10  (1.45–4.25) | 3.34  (2.16–4.56) | 3.00  (2.07–3.99) | 2.80  (2.53–3.00) |
| **Body length** | Anterior margin of the head to the apex of the abdomen | 14.36  (10.54–17.31) | 14.64  (10.91–17.00) | 13.78  (10.41–16.02) | 13.15  (10.87–15.49) |
| **Wing length** | Base of the fore wing at the tegula to the apex of the wing | 11.67  (8.95–13.97) | 11.64  (9.30–13.12) | 11.25  (8.34–13.14) | 10.46  (8.63–11.47) |
| **Abdomen width** | Width at the anterior portion of the gaster | 2.90  (2.07–3.60) | 3.00  (2.34–3.74) | 2.84  (2.08–3.56) | 2.81  (2.44–3.35) |
| **PC1** | Principle component 1 | 0.30  (−5.90–4.68) | 0.95  (−3.95–4.25) | −0.50  (−5.93–3.19) | −1.66  (−4.18–1.09) |
