# Supplementary material for: Sex, density dependence, and urbanization level shape host infection by an obligate endoparasite
Source: PLoS One. 2026 Feb 12;21(2):e0340623. doi: 10.1371/journal.pone.0340623 (PMC12900303; doi:10.1371/journal.pone.0340623)
Supplement: S2 Table — All traits show moderate to high positive correlation. (DOCX) [file pone.0340623.s002.docx]

Table S2. Pearson correlation coefficients among morphological traits of *Isodontia* *mexicana*. All traits show moderate to high positive correlation.

|  | Head width | Head length | Body length | Wing length | Abdomen width |
| --- | --- | --- | --- | --- | --- |
| Head width | 1 | 0.6757234 | 0.7623699 | 0.8056653 | 0.7400907 |
| Head length | 0.6757234 | 1 | 0.6352518 | 0.6715356 | 0.5407501 |
| Body length | 0.7623699 | 0.6352518 | 1 | 0.8440334 | 0.6615856 |
| Wing length | 0.8056653 | 0.6715356 | 0.8440334 | 1 | 0.7638419 |
| Abdomen width | 0.7400907 | 0.5407501 | 0.6615856 | 0.7638419 | 1 |
