# Supplementary material for: Sex, density dependence, and urbanization level shape host infection by an obligate endoparasite
Source: PLoS One. 2026 Feb 12;21(2):e0340623. doi: 10.1371/journal.pone.0340623 (PMC12900303; doi:10.1371/journal.pone.0340623)
Supplement: S3 Table — The top section shows the standard deviation, proportion of variance explained, and cumulative variance for the first five principal components. The lower section lists the loadings (eigenvectors) of each trait on each principal component. (DOCX) [file pone.0340623.s003.docx]

Table S3. Principal component analysis (PCA) of five morphological traits measured in *Isodontia mexicana*. The top section shows the standard deviation, proportion of variance explained, and cumulative variance for the first five principal components. The lower section lists the loadings (eigenvectors) of each trait on each principal component.

|  | PC1 | PC2 | PC3 | PC4 | PC5 |
| --- | --- | --- | --- | --- | --- |
| Standard deviation | 1.97 | 0.65786 | 0.57245 | 0.46419 | 0.37942 |
| Proportion of variance | 0.776 | 0.08656 | 0.06554 | 0.04309 | 0.02879 |
| Cumulative proportion | 0.776 | 0.86258 | 0.92811 | 0.97121 | 1 |
| Head width | 0.4620098 | 0.06312425 | -0.1169971 | 0.8760823 | 0.03679258 |
| Head length | 0.4110149 | -0.8559811 | -0.2411079 | -0.1899772 | 0.06433334 |
| Body length | 0.4539484 | 0.11480072 | 0.6595896 | -0.1830452 | 0.55873757 |
| Wing length | 0.4725261 | 0.12879268 | 0.2812814 | -0.1871527 | -0.8037327 |
| Abdomen width | 0.4338934 | 0.48326492 | -0.6434292 | -0.3575706 | 0.19061393 |
