# Supplementary material for: Sex, density dependence, and urbanization level shape host infection by an obligate endoparasite
Source: PLoS One. 2026 Feb 12;21(2):e0340623. doi: 10.1371/journal.pone.0340623 (PMC12900303; doi:10.1371/journal.pone.0340623)
Supplement: S4 Table — Tree cover is strongly negatively correlated with urban cover, while grass cover shows weak correlations with both. (DOCX) [file pone.0340623.s004.docx]

Table S4. Pearson correlation coefficients among land cover variables within a 250 m buffer around study sites. Tree cover is strongly negatively correlated with urban cover, while grass cover shows weak correlations with both.

|  | % Tree cover | % Open green cover | % Urban cover |
| --- | --- | --- | --- |
| % Tree cover | 1 | -0.3885233 | -0.877987 |
| % Open green cover | -0.3885233 | 1 | -0.0994412 |
| % Urban cover | -0.877987 | -0.0994412 | 1 |
