# Supplementary material for: Sex, density dependence, and urbanization level shape host infection by an obligate endoparasite
Source: PLoS One. 2026 Feb 12;21(2):e0340623. doi: 10.1371/journal.pone.0340623 (PMC12900303; doi:10.1371/journal.pone.0340623)
Supplement: S5 Table — The top section shows the standard deviation, proportion of variance, and cumulative variance explained by each principal component. The lower section shows the loadings (eigenvectors) of each variable on the first three principal components. (DOCX) [file pone.0340623.s005.docx]

Table S5. Summary of principal component analysis (PCA) results based on three land cover variables within a 250 m buffer. The top section shows the standard deviation, proportion of variance, and cumulative variance explained by each principal component. The lower section shows the loadings (eigenvectors) of each variable on the first three principal components.

|  | PC1 | PC2 | PC3 |
| --- | --- | --- | --- |
| Standard deviation | 1.3789 | 1.048 | 0.01742 |
| Proportion of variance | 0.6338 | 0.3661 | 0.0001 |
| Cumulative proportion | 0.6338 | 0.9999 | 1 |
| % Tree cover | 0.7178106 | 0.1353917 | 0.6829473 |
| % Open green cover | -0.1249284 | -0.9399435 | 0.3176461 |
| % Urban cover | -0.6849385 | 0.3133292 | 0.6577872 |
