# Supplementary material for: Sex, density dependence, and urbanization level shape host infection by an obligate endoparasite
Source: PLoS One. 2026 Feb 12;21(2):e0340623. doi: 10.1371/journal.pone.0340623 (PMC12900303; doi:10.1371/journal.pone.0340623)
Supplement: S1 Fig — (DOCX) [file pone.0340623.s006.docx]

**Fig S1.** Principal component analysis (PCA) biplot showing variation in landscape cover across sampling sites.
