# Supplementary material for: Sex, density dependence, and urbanization level shape host infection by an obligate endoparasite
Source: PLoS One. 2026 Feb 12;21(2):e0340623. doi: 10.1371/journal.pone.0340623 (PMC12900303; doi:10.1371/journal.pone.0340623)
Supplement: S2 Fig — (DOCX) [file pone.0340623.s007.docx]

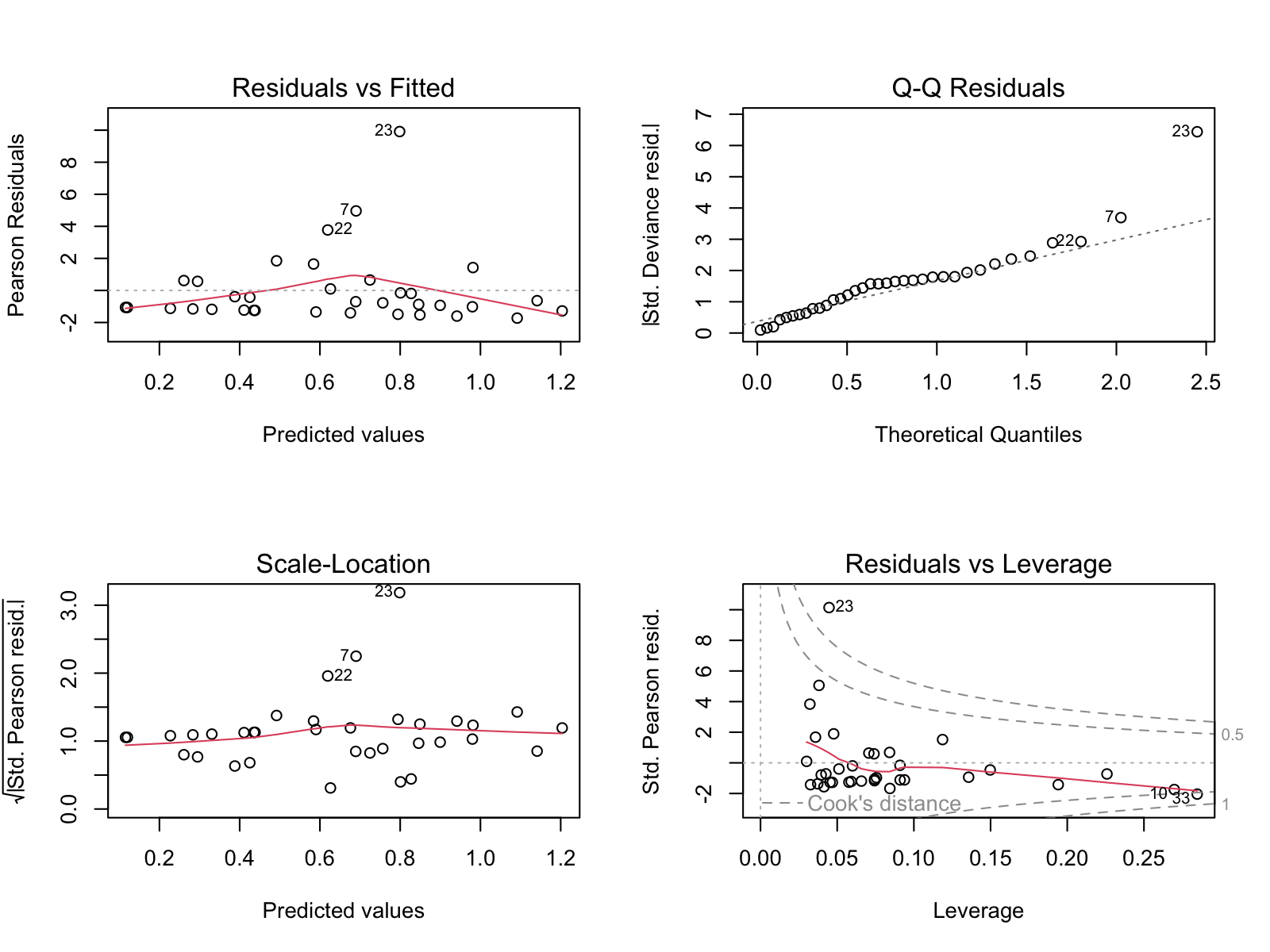


**Fig. S2.** Diagnostic plots for the Poisson GLM testing effects of landscape composition (PC1, PC2) on I. mexicana abundance.
