# Supplementary material for: Sex, density dependence, and urbanization level shape host infection by an obligate endoparasite
Source: PLoS One. 2026 Feb 12;21(2):e0340623. doi: 10.1371/journal.pone.0340623 (PMC12900303; doi:10.1371/journal.pone.0340623)
Supplement: S1 File — (ZIP) [file pone.0340623.s011.zip › Data/MacIvor and Hall Data README file.docx]

Notes on the column headings used in the attached data to: MacIvor and Hall “Sex, density dependence, and urbanization level shape host infection by an obligate endoparasite” published in PLoS One.

The data used in this paper are in three .csv files: iso_full_final.csv, land_iso_final.csv, stylop_final.csv. iso_full_final.csv contains data recorded at the individual level, including morphometrics (in mm) and stylopization. land_iso_final.csv includes land cover classification and site type recorded at the site level. stylop_final.csv includes information on the strepsipterans recorded from each wasp. These three .csv files are connected via columns SiteCode (*i.e.,* site) and Wasp (*i.e.,* individual *Isodontia mexicana* wasp inspected).

| **Column name** | **Data type** | **Notes** |
| --- | --- | --- |
| Year | Time | Year of trap nest deployment to survey wasps. |
| SiteCode | Category | Each site is identified using a unique five-digit code. |
| Wasp | Sampling unit | An individual wasp recovered from a trap nest and inspected for this study. |
| Sex | Binary | Recorded whether the wasp was male or female. |
| Head_width | Continuous | measured with digital caliper in mm. |
| Body_length | Continuous | measured with digital caliper in mm. |
| Head_length | Continuous | measured with digital caliper in mm. |
| Wing_length | Continuous | measured with digital caliper in mm. |
| Abd_width | Continuous | measured with digital caliper in mm. |
| strep | Count | Abundance of strepsipteran identified from a single wasp adult. |
| para | Binary | Whether a wasp was stylopized (yes or no). |

**iso_full_final.csv**

**land_iso_final.csv**

| **Column name** | **Data type** | **Notes** |
| --- | --- | --- |
| SiteCode | Category | Each site is identified using a unique five-digit code. |
| Type | Category | Green space type: Garden, Park, Community Garden, Green Roof. |
| perc_tree_250 | Proportion | Percent tree cover (representing treed canopy cover) within a 250m radius around a site. |
| perc_grass_250 | Proportion | Percent grass cover (representing open green space) within a 250m radius around a site. |
| perc_urb_250 | Proportion | Percent tree cover within a 250m radius around a site. |

**stylop_final.csv**

| **Column name** | **Data type** | **Notes** |
| --- | --- | --- |
| Year | Time | Year of trap nest deployment to survey wasps. |
| SiteCode | Category | Each site is identified using a unique five-digit code. |
| Wasp | Category | An individual stylopized wasp examined in this study. |
| Sex | Binary | Recorded whether the wasp was male or female. |
| Head_width | Continuous | measured with digital caliper in mm. |
| Body_length | Continuous | measured with digital caliper in mm. |
| Head_length | Continuous | measured with digital caliper in mm. |
| Wing_length | Continuous | measured with digital caliper in mm. |
| Abd_width | Continuous | measured with digital caliper in mm. |
| # Strep | Count | Number of male strepsipterans recorded from the stylopized wasp. |
| Strep | Sampling Unit | An individual strepsipteran recovered from a stylopized wasp and inspected for this study. |
| emerged | Binary | Did males had eclosed, leaving empty puparia (yes or no). |
| Left_right | left/right | Location of the strepsipteran. Left or right side of the abdomen. |
| Terg_stern | tergite/sternite | Location of the strepsipteran. Top or bottom of the abdomen. |
| Seg_num | count | Which abdominal segment strepsipteran was observed (front to back). |
| Note | - | Any additional notes recorded. |
